# Smart Medical Evacuation Support System for the Military

**DOI:** 10.3390/s24144581

**Published:** 2024-07-15

**Authors:** Jaroslaw Krygier, Piotr Lubkowski, Krzysztof Maslanka, Andrzej P. Dobrowolski, Tomasz Mrozek, Wojciech Znaniecki, Pawel Oskwarek

**Affiliations:** 1 Faculty of Electronics, Institute of Communications Systems, Military University of Technology, gen. Sylwester Kaliski Str. No. 2, 00-908 Warsaw, Poland; piotr.lubkowski@wat.edu.pl (P.L.); krzysztof.maslanka@wat.edu.pl (K.M.); andrzej.dobrowolski@wat.edu.pl (A.P.D.); tomasz.mrozek@wat.edu.pl (T.M.); 2 TELDAT Sp. z o.o. sp.k, Cicha 19-27, 85-650 Bydgoszcz, Poland; wznaniecki@teldat.com.pl; 3 Military Institute of Medicine—National Research Institute, Szaserow 128, 04-141 Warsaw, Poland; poskwarek@wim.mil.pl

**Keywords:** medical triage, medical evacuation, biomedical signals, vital signs, decision support system

## Abstract

Medical support in crisis situations is a major challenge. Efficient implementation of the medical evacuation process especially in operations with limited human resources that may occur during armed conflicts can limit the loss of these resources. Proper evacuation of wounded soldiers from the battlefield can increase the chances of their survival and rapid return to further military operations. This paper presents the technical details of the decision support system for medical evacuation to support this process. The basis for the functioning of this system is the continuous measurement of vital signs of soldiers via a specialized measurement module with a set of medical sensors. Vital signs values are then transmitted via the communication module to the analysis and inference module, which automatically determines the color of medical triage and the soldier’s chance of survival. This paper presents the results of tests of our system to validate it, which were carried out using test vectors of soldiers’ vital signs, as well as the results of the system’s performance on a group of volunteers who performed typical activities of tactical operations. The results of this study showed the usefulness of the developed system for supporting military medical services in military operations.

## 1. Introduction

Medical evacuation is an important part of operation in situations where human life is at risk. Various teams of people participating in rescue operations, military missions, or police prevention activities risk their health and lives to save injured or endangered people. In a typical situation, information about an incident that threatens the health of a team member is relayed by another person on the team, who informs the personnel in charge of medical evacuation of the possible need for evacuation.

Currently developed systems for monitoring human vital signs do not allow monitoring and supporting activities for the whole of society but allow equipping teams of people involved in situations that may threaten their health and life. Imagine a situation where the firefighters that are taking part in a rescue operation, a police officer that is pursuing dangerous criminals, or soldiers that are taking part in warfare are equipped with a system that allows them to assess their health and alert the medical emergency team of a situation that threatens their health and evacuation necessitates. Such an alarm may also be generated in a situation when several members of the team involved in the operation are injured and there is no way to inform medical personnel to evacuate. In addition, a system of this type equipped with intelligent algorithms will allow automatic selection of at-risk patients (named in medical language as a medical triage), which will allow an appropriate decision to be made on the order of evacuation of individual patients from the scene of an accident or battlefield.

Current triage procedures require medical personnel to be present at the scene of an accident to decide the order of evacuation based on the injuries of individual patients. Such a procedure requires the involvement of a significant number of medical personnel (paramedics and rescue doctors) on the scene, making its effectiveness low, especially in the case of a large number of victims, which is particularly important in military operations.

This article presents an intelligent system to autonomously assess the condition of the injured and to support the medical evacuation (MEDEVAC) team in the process of evacuating the injured from the scene of the incident. Since the presented system supports medical personnel’s evacuation decisions, we called it the decision support system for medical evacuation (DSS-MEDEVAC). The DSS-MEDEVAC system was developed primarily to support military medical evacuation teams on the battlefield. However, its elements can be successfully used in non-military rescue operations, where the victims of accidents may be medically monitored people (such as in firefighting operations, large-scale police operations, and others). The main capabilities of an early version of DSS-MEDEVAC and the first results of its laboratory operation are presented in [1]. In this article, the current version of the system verified with field tests is described, as well as the results of tests conducted on soldiers performing typical combat tasks and soldiers simulating wounded victims (to the extent feasible without risk to the health of soldiers). In order to force the system to respond appropriately to wounds that qualify it to alert medical personnel to the need for immediate evacuation in the event of a life-threatening casualty, a series of test vectors were prepared to simulate critical human vital signs during an accident.

The basic part of the DSS-MEDEVAC system is the measurement module that is responsible for constant monitoring of vital signs. Each monitored person (soldier on the battlefield, firefighter, or member of a rescue and search group taking part in a rescue operation) is equipped with measurement sensors that do not restrict movement of the person and record basic vital signs (heart rate, systolic blood pressure, oxygen saturation respiratory rate, physical activity, body position, and skin temperature). These signs are sent with a specific frequency to the management center, where they are processed, and the patient’s health status is assessed. In the event of a critical situation, requiring the emergency intervention of a medical evacuation team, the medical personnel operating the system are informed in the form of an alarm about the detected threat. This alarm is generated based on the analysis of vital signs, evacuation order decisions, and a novel method proposed by the authors that determines the probability of survival of the victim. Medical staff operating the battlefield medical monitoring center can assess the patient’s health status based on his vital signs, but at the same time can observe biomedical signals (e.g., electrocardiogram, ECG, and photoplethysmogram, PPG) from the medical sensors which can be sent to the management center on demand. These signals will allow for additional assessment of the patient’s condition by the medic operating the system, enabling final decision-making based on the necessity and order of evacuation of the injured.

The DSS-MEDEVAC system developed by the authors of this paper provides mechanisms for intelligent, continuous assessment of soldiers’ health status and supports the process of medical evacuation under combat conditions, using advanced decision-making algorithms to prioritize the evacuation of the wounded. Compared to the solutions analyzed in Section 2 or described in [1], the DSS-MEDEVAC system is distinguished by several key features:

- Integration with military systems: the DSS-MEDEVAC system is designed to operate under specific combat conditions, taking into account communication and environmental constraints that are not present in civilian medical systems;
- Advanced triage algorithms: the DSS-MEDEVAC system uses advanced algorithms based on multiple vital signs (mainly heart rate—HR, respiratory rate—RR, oxygen saturation of the blood—SpO_2_, and systolic blood pressure—SBP) that are dynamically assessed and updated in real time, allowing for precise medical triage. In addition, the chances of survival function proposed by the authors of this paper develops the possibility of faster and more accurate triage, which is always a challenge for medics arriving at the scene after some time. The chances of survival function, already at the stage of arriving at the scene, prioritizes the wounded soldiers, enabling the arriving medics, in a shorter period of time, to make the most accurate decisions on the condition and triage of the soldiers;
- The developed system, or more precisely, its individual components, such as portable measurement modules, provide high mobility and autonomy, which is crucial in dynamically changing battlefield conditions.
- The main contributions of this paper are as follows:
- Description of a complete, field-tested system for decision support of medical evacuation of casualties (soldiers);
- Pre-screening of the algorithm for assessing the health status of the monitored soldier;
- Presentation of the author’s method for assessing the chances of survival of a wounded soldier and using this knowledge to indicate the order of medical evacuation;
- Description of the results of using the system in field tests.

The article is organized as follows. Section 2 indicates the result of the analysis of similar systems and methods used to assess the health of monitored patients in order to develop a decision on the need for possible medical evacuation. Section 3 discusses the architecture of the system. In Section 4, the conditions of the system’s field tests are clarified. In addition, it includes a discussion of the results of testing the DSS-MEDEVAC system with test vectors and using soldiers performing combat operations. A summary and directions for further work are provided in Section 5.

## 2. Related Work

In recent years, there have been significant developments in technologies supporting battlefield medicine, particularly systems based on artificial intelligence (AI) and advanced decision-making algorithms. The goal of these technologies is to improve triage processes, medical evacuation, and overall management of medical care in combat conditions. In this section, we will discuss current solutions used worldwide and analyze the innovation of the DSS-MEDEVAC system against existing technologies.

Solutions have long been sought to optimize and accelerate the performance of medics on the battlefield, especially in an incident with a large number of casualties. The use of modern technology is also applicable to the medical triage in the theater of operations. In the specialized literature, we can find attempts to support the triage in both battlefield and civilian medicine.

DARPA’s “In the Moment” (ITM) program [2] focuses on developing AI systems capable of autonomous medical decision-making similar to that of experienced medics. These systems are designed to support small military units during triage and mass casualty management. ITM algorithms are designed to be able to autonomously assess the health of soldiers and prioritize medical evacuation.

Another project dealing with triage support includes ATRACT (A Trustworthy Robotic Autonomous system to support Casualty Triage) [3], which aims to create autonomous drones to assist triage and evacuation of the injured in the crucial minutes after an injury. These drones, equipped with advanced sensors and AI algorithms, can quickly and accurately assess injuries and provide necessary medical information to emergency personnel. This approach minimizes response time and increases the chances of surviving for the wounded on the battlefield, but it still does not provide the ability to monitor a soldier in real time, to assess his state of health, even before the stage of an incident resulting in the need for medical assistance, including emergency medical evacuation.

The civilian medical community is also trying to implement new technologies to optimize the performance of triage processes. Studies have shown that AI algorithms can effectively predict the need for intensive care, outperforming traditional patient assessment methods. For example, a study published in *Intensive Care* [4] found that AI models, such as deep neural networks and gradient boosted decision trees, outperformed traditional triage methods in assessing clinical outcomes.

The DSS-MEDEVAC system stands out from other systems due to its integration of advanced decision-making algorithms and AI technology, designed specifically for military applications. Our system monitors multiple vital signs, such as HR, RR, and SpO_2_SBP, and thus dynamically assesses soldiers’ health status in real time. This makes it possible to accurately prioritize evacuation and automatically generate alerts when health risks are detected. Many studies can be found in the literature on the use of advanced algorithms to analyze the condition of the injured as well as triage systems that attempt to use artificial intelligence. Of particular recent interest is the use of cutting-edge technology that can be used in real-time triage optimization. Shaoxiong Sun et al., in [5], explore the possibility of estimating systolic blood pressure using ECG and PPG signals. The authors show how biomedical signal analysis algorithms can be used to accurately monitor patients’ vital signs, which is crucial in the context of our medical evacuation decision support system. Algorithms discussed in [5] indicate the validity of using basic vital signs measurements in our system to assess the health of soldiers during military operations and make evacuation decisions when necessary.

Rastegar S. et al., in [6], present a hybrid model based on convolutional neural networks (CNNs) and support vector regression (SVR) for blood pressure estimation. It means that the integration of advanced artificial intelligence methods with classical data analysis techniques provides a solid basis for implementation in medical systems such as our DSS-MEDEVAC.

Ma G. et al., in [7], focus on evaluating and visualizing the contribution of characteristic ECG waveforms in PPG-based blood pressure estimation. This study underscores the importance of precise analysis of biomedical signals and their application in health monitoring systems, which is crucial to remote health monitoring.

Many authors focus on the aspect of blood pressure measurement and its impact on assessing the health risks of monitored patients. The paper [8] by Asmar S. et al., for example, analyses the relationship between systolic blood pressure and brain injury in the context of emergency departments. The findings are important for our system, which needs to quickly and accurately assess the condition of casualties on the battlefield, where brain injuries can be a common occurrence. Hence, one of the future works connected with our system is the use of bispectral measurement electrodes (BISs), as suggested in [8].

Health monitoring systems for civilians are numerous and new ones are constantly being developed. Nevertheless, it should be noted that civilian vital signs monitoring systems available on the market mainly focus on monitoring previously diagnosed patients in hospital or home settings, using technologies such as telemetry, wearables, and mobile apps for remote health monitoring. Examples of such systems include the following: Philips IntelliVue [9,10], a patient monitoring system for hospitals that offers monitoring of ECG, blood pressure, saturation, respiratory rate, and other parameters; Fitbit health monitoring devices [11,12], a wearable device that monitors physical activity, heart rate, sleep quality, and other health indicators, often used for remote health monitoring; and iRhythm Zio Patch [13,14], a wearable long-term ECG monitoring device used to diagnose heart rhythm disorders in the home environment. Our DSS-MEDEVAC system, developed specifically for military use, is distinguished from the civilian systems indicated above by several key features. First, resilience to extreme conditions. Our system is designed to operate in harsh field conditions, such as extreme temperatures, humidity, dust, and vibration, which is crucial on the battlefield. Civilian systems, such as Philips IntelliVue, are mainly designed for stable hospital conditions. Second, integration with command-and-control systems. Our system is fully integrated with military command and control systems, allowing for the tracking of the health of thousands of soldiers in real time and suggesting evacuation decisions based on ongoing measurements of soldiers’ vital signs. Civilian systems, such as Fitbit Health Solutions, typically operate autonomously and are not part of larger emergency management systems. Third, augmented situational awareness. Our system uses advanced artificial intelligence algorithms to analyze data from a variety of sources, such as biomedical sensors, GPS location, and physical activity sensors, allowing for more precise and rapid decision-making about the need to evacuate soldiers from the battlefield. Civilian systems, such as iRhythm Zio Patch, are mainly focused on monitoring single health parameters.

A large number of other references for many solutions of decision support systems during medical triage have been described in [1]; hence, this article does not revisit them. The rationale for the selection of a number of vital signs necessary for monitoring in order to develop a decision on triage during medical evacuation was also indicated there.

As the papers cited above show, significant progress has been made in recent years in the use of AI in the medical triage in both combat and civilian settings. Projects such as DARPA ITM and ATRACT, as well as applications of AI in civilian hospitals, demonstrate how technology can support medics in making critical decisions and managing medical resources. The DSS-MEDEVAC system is part of this trend, offering advanced solutions dedicated to specific military needs, making it a unique and extremely valuable tool in battlefield medicine. However, compared to the aforementioned systems, our system offers unique solutions tailored to specific combat conditions. While ITM and ATRACT focus on autonomous decision-making and the use of drones, DSS-MEDEVAC integrates these technologies into a more complex battlefield medical care management system. In addition, it uses advanced communication mechanisms and data transmission protocols to ensure reliable operation in harsh combat environments. Moreover, DSS-MEDEVAC can be used not only in military operations, but also in rescue and prevention operations, such as firefighting and police operations. Thanks to the system’s flexibility and scalability, it can be used in a variety of scenarios where quick and accurate health assessment and evacuation are crucial to saving lives.

## 3. Architecture of the DSS-MEDEVAC System

### 3.1. System Overview

Typical healthcare telemonitoring systems are designed to continuously monitor specific, usually single, vital signs of patients, on the basis of which it is possible to administer specific medications or respond in a health-threatening, less frequently life-threatening situation. In the case of a system designed to support the evacuation of soldiers from the battlefield, it is necessary to monitor many vital signs simultaneously and, based on the measured values, determine triage values for each monitored soldier using the system’s intelligent algorithms. Automated triage of casualties is particularly important for military operations, where it is assumed that every soldier involved in battlefield operations will be monitored. This requires reliable measurement of specific vital signs of each monitored soldier and simultaneous transmission of the measurement results to the Battlefield Management System (BMS). It should be noted that the vital signs of soldiers can be measured while they are performing intense activities in stressful situations. In commercial systems, the operating conditions of the health monitoring system are completely different, and at the same time, data can be sent via broadband Internet connections, which is unattainable in tactical communication systems.

The main abbreviations used in this paper are collected in Abbreviations at the end of the paper.

The main requirements for the DSS-MEDEVAC system are presented in [1] where the first version of the system, tested under laboratory conditions, is described. Based on the requirements, the system’s service architecture was developed and presented in accordance with NATO Architecture Framework version 4 (NAFv4) [15]. In order to maintain the consistency of this paper, the requirements on the system will be briefly indicated here. The basic requirement for the system is the need to measure, record, and present current information of soldier’s vital signs and a preliminary triage decision for medical personnel and selected commanders. The system must also process the collected information, and if the analysis and inference capability (AIC) module detects a life-threatening condition, it must alert medical personnel about such a situation. The triage is based on the following measured human vital signs that are sufficient for analysis: SpO_2_, HR, SBP, RR, mean physical activity (PA), and body position (BP). In addition, each of the measured vital sign is assessed with developed algorithms to determine the quality of its measurement, and calibration and reference data are entered for each soldier.

Another important requirement on the DSS-MEDEVAC system is the ability to view biomedical signals recorded via the personal measurement module (Measurement Subsystem) that is equipped with a set of medical sensors. This solution allows medical personnel to observe alerted soldiers and determine their current health status based on biomedical signals such as: ECG, PPG, physical activity signal (PAS), and respiratory rate signal (RES). At the same time, due to the military purpose of the system, the medical parameters of a wounded soldier for whom a decision on the need for necessary medical evacuation has been made, along with other required data in accordance with Tactical Field Care procedures, are saved to the electronic version of the Tactical Combat Casualty Care Card (TCCC or TC3 Card) [16].

**Figure 1:**
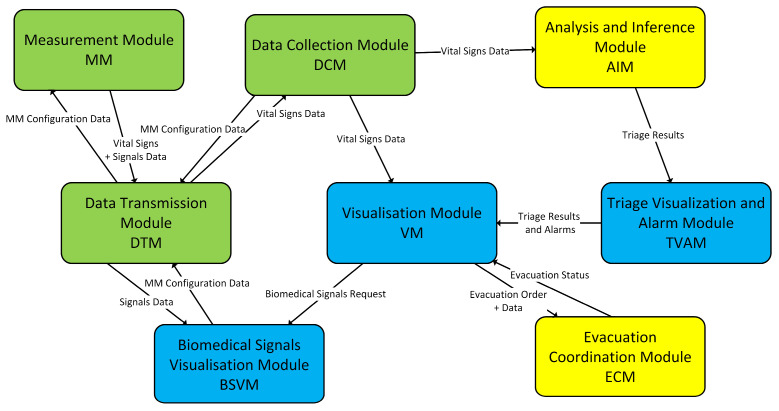
Functional architecture of the DSS-MEDEVAC system.

The functional architecture of the DSS-MEDEVAC system is shown in Figure 1. Measurement of vital signs is performed via the measurement module (MM). This module receives a request from the Data Collection Module (DCM), which specifies the vital signs that will be recorded during system operation (MM Configuration Data). The recorded data (vital signs + signals data) are sent to the DCM, where they are stored in a database associated with the personal and calibration data of the monitored person. At the same time, the data go to the Visualization Module (VM) for display in the Medical Support Group (MSG) Portal and to the Analysis and Inference Module (AIM). The AIM continuously analyzes the received vital sings values and assesses the health and life-threatening status of all monitored patients. The results of this analysis (Triage Result) are sent to the Triage Visualization and Alarm Module (TVAM), which presents them in the VM in the form of an appropriate color code for each patient (Triage Results and Alarms). At the same time, the TVAM detects triage values indicating a threat state and alerts medical personnel when such an event occurs. Analysis of the soldier’s health condition is also possible by observing biomedical signals recorded with MM sensors at the request of the MSG Portal operator. Then, the data recorded via the sensor specified in the configuration (Signals Data) are sent directly to the Biomedical Signals Visualization Module (BSVM) and are presented in the portal in the form of waveforms of values of the recorded biomedical signal samples.

**Figure 2:**
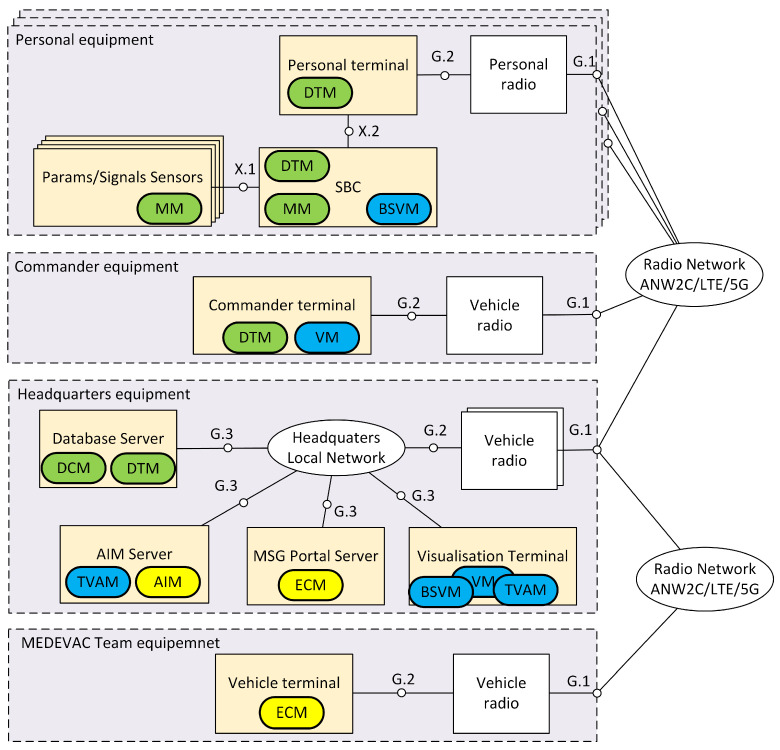
Physical architecture of the DSS-MEDEVAC system (the colors indicate: gray—groups of equipment; also indicated by dashed lines, gold—hardware components, white—components of the data transmission network, green—components responsible for data collection and delivery, dark yellow—components responsible for data analysis, blue—components responsible for data visualization; solid lines connect the interfaces).

The physical architecture and distribution of functional modules across the physical components of the system are shown in Figure 2. The system can be divided into four basic elements:

- Personal equipment—hardware components included in the soldier’s personal equipment;
- Commander’s equipment—hardware devices that are part of the commander’s equipment;
- Headquarters equipment—equipment of the management center;
- MEDEVAC team equipment—hardware components of the medical evacuation teams.

The measurement components of the system are the basic equipment of the monitored soldier. The system’s sensors are connected to a single-board computer (SBC) via an X.1 internal interface, which processes the collected data and, upon request from the management center, sends it to the soldier’s personal terminal via the X.2 interface. This interface uses a Universal Serial Bus (USB) port and the DSS-MEDEVAC Data Transmission Protocol (MDTP) prepared for the need of the reliable control of the sensors’ data transmission. A microservice is running in the personal terminal, allowing the transfer of data, both vital signs and biomedical signals, using an appropriate Google Remote Procedure Calls (GRPC) to the management center. Vital signs values are sent using the battlefield management system’s built-in mechanisms required for reliable data delivery. The data are compressed to minimize transmitted data streams. When requested, the data stream of signals is sent using typical streaming data transmission to the BSVM—but using the same radio network.

The data registered via the management module are stored and processed in servers located in the management center. For the primary use of the system, which is to support the management of medical evacuation on the battlefield, the center will be located at the brigade headquarters. There are three primary servers responsible for storing data and processing it to determine the triage values for individual soldier. They are the Database Server, AIM Server, and MSG Portal Server. From the medical portal, an order is also sent to the MEDEVAC team. The MEDEVAC team also has access to information initially filled in the TC3 card associated with a wounded soldier. The use of a tactical communications system (personal and mobile radios) is realized through a G.2 interface, which is usually the Ethernet-based interface. The tactical communications network can be based on any technology used by the military, including radio Advanced Networking Wideband Waveform (ANW2C), the military version of the Long-Term Evolution (LTE) technique or the military version of the 5th Generation (5G) mobile networks. Communication between devices in the management center will be performed via the Ethernet network.

### 3.2. Measurement Module

The basic task of the measurement module is to measure the patient’s vital sings using appropriate medical sensors. Sensors that perform such measurements are commonly used in civilian medical systems. However, in these systems the measurement is usually performed in a prepared static position of the patient. Providing such conditions in tactical systems is not possible. The soldier will be constantly moving, making various, often violent movements, and his muscle tension will be constantly changing. Moreover, in the conditions in which the measurement module is to be used, there may be situations in which individual sensors may be destroyed, damaged, or disconnected as a result of tactical actions or even during wounds. Hence, there is the need to develop the measurement module and its processing algorithms in such a way that these types of situations can be considered.

During the development of the measurement module, it was important to select an appropriate set of parameters whose values will be measured so that they would be useful from the point of view of the Analysis and Inference Module. The list of selected vital signs is presented in Table 1. An extensive justification for the choice of these parameters is contained in [1], and hence is not described in this paper. The total size of the data required to encode all vital signs are 18 Bytes. Vital signs are calculated based on biomedical signals recorded with individual sensors. Thus, for example, HR is determined from the recorded ECG biomedical signal, but SBP is determined using both the ECG and PPG biomedical signals. Medical sensors, whose abbreviations are indicated in the last column of Table 1, are responsible for recording individual biomedical signals.

**Figure 3:**
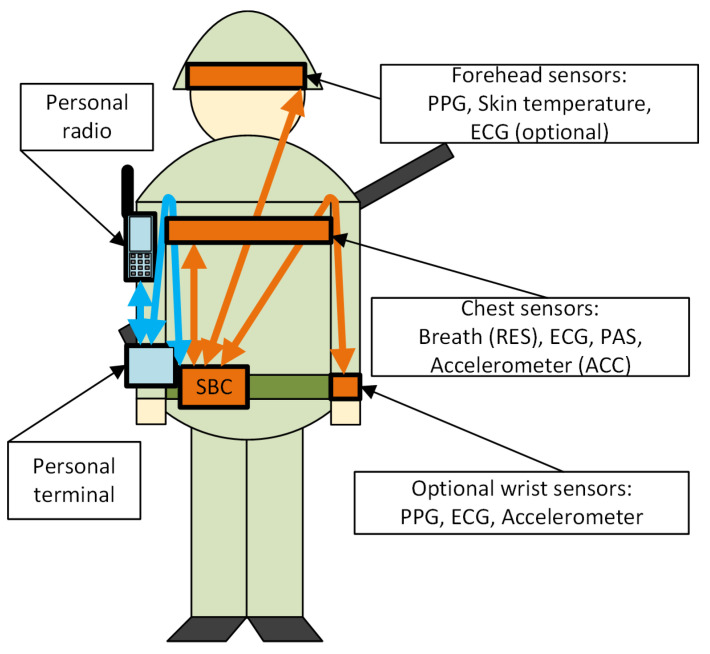
Arrangement of the MM sensors and their integration with the personal equipment of the monitored soldier.

Values of vital signs are averaged and sent to the personal terminal every 1 s. The vital signs themselves are determined from biomedical signals measured via the sensors located on the forehead, chest, and optionally on the wrist of a soldier. Typically, they are integrated with a soldier’s helmet and with protective clothing or uniforms (Figure 3).

Biomedical signals measured with the sensors are listed in Table 2. The table also shows the sampling frequencies used for the measured biomedical signals and the data transmission rates corresponding to the indicated sampling frequencies.

Values of vital signs calculated with the measurement module on the basis of recorded biomedical signals and sent to the personal terminal are forwarded to the Analysis and Inference Modules at a reduced rate in order to reduce the occupation of radio resources of the tactical communications system. Currently, these vital signs values are sent to the AIM every 1 min, forcing data transfer at a rate of 2400 bit/s.

The RR is measured using sensors in a measuring belt placed on the chest. Two angular strain sensors (SparkFun SEN-08606 and SparkFun SEN-10264, Niwot, CO, USA) are placed in the measuring belt on either side of the chest position and a pressure sensor (DFRobot SEN0293, Shanghai, China) is placed on the left side. Our system uses 2 ECG measurement paths in parallel (in the forehead and chest belt). Each track uses three electrodes—two active and one feedback—attached to the AFE MAX30003 chipset (Analog Devices, Wilmington, NC, USA). R-waves are detected from the ECG signal and then HR is calculated. The system is also equipped with 2 PPG sensors. Each has 3 measurement channels for red, green, infrared color LEDs, respectively. The sensors are placed in the center of the forehead and optionally on the wrist. From the PPG signal, HR and SpO_2_ are calculated. In addition, body temperature is measured using the TMP117 High-Accuracy, Low-Power, Digital Temperature Sensor, and the soldier’s body position and physical activity is measured using the MC3479 3-Axis accelerometer. In the current version of our system, the SBC with wiring weighs 162 g, the chest strap weighs 171 g, and the forehead band weighs 92 g. However, it should be noted that there is work on further miniaturization of the individual components of the measurement module. Detailed ways of using individual sensors and methods of determining vital signs from biomedical signals that were recorded in the measurement module of our system are described in articles published by our project team, in particular in [17,18,19,20].

**Figure 4:**
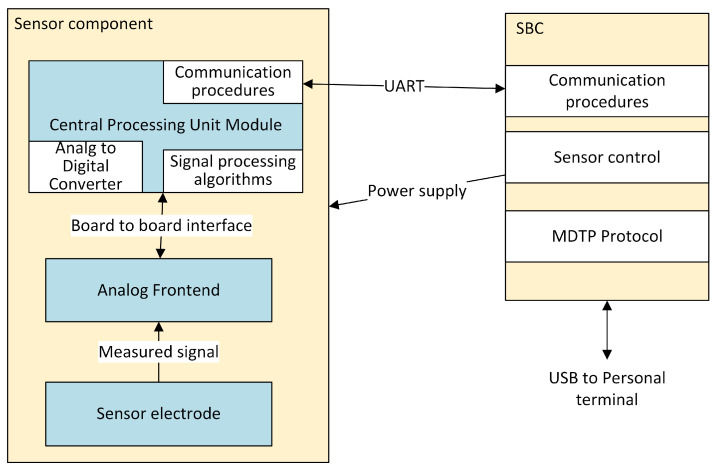
Structure of DSS-MEDEVAC sensor.

The measurement module has a modular structure allowing for easy expansion with additional sensors. The structure of the sensors used in our system is shown in Figure 4. The single-board computer (SBC) is an integrator of sensors. It receives and processes data from sensors and is built based on a microcontroller with ARM architecture (ARM Cortex-M7/STM32H7A3VI/—STMicroelectronics, Plan-les-Ouates, Switzerland—process with 280 MHz CPU and 2 MB Flash). Data from the sensors are sent to the SBC via the Universal Asynchronous Receiver–Transmitter (UART) bus. In the sensor, analog signals are filtered and amplified into an analog front end (AFE), then converted into digital form and passed on for further processing to the SBC. A very important element is the time synchronization between individual sensors, which will enable the measurement of the SBP parameter based on the propagation time (PAT—Pulse Arrival Time or Pulse Transit Time) of the pulse wave calculated from ECG/PPG signals.

Sensors are synchronized with the SBC by periodically sending requests to all sensors, which should respond within a certain fixed time, shorter than the highest sampling period of the selected sensor (e.g., for ECG the response time must be less than 4 ms). Thanks to the priority handling of SBC interruptions from the sensor’s microcontroller, the response is sent with a very low delay (of the order of 100 µs), which allows the sensors to synchronize their timing precisely.

### 3.3. Data Transmission Module

The main goal of the data transmission module is to provide the DSS-MEDEVAC system with a service enabling reliable and safe data exchange between its modules. The data transmission system itself in the military option is based on the radio battlefield network, but it can also use other available transmission systems, such as, for example, 4G/5G cellular communication systems, WiFi wireless communication systems, or others. Aggregation of vital sign values measured in the measurement module is carried out via the Headquarter Management System (HMS), developed to support Command, Control, Communication, Intelligence, and Surveillance (C3IS) capability during military operations [21,22].

**Figure 5:**
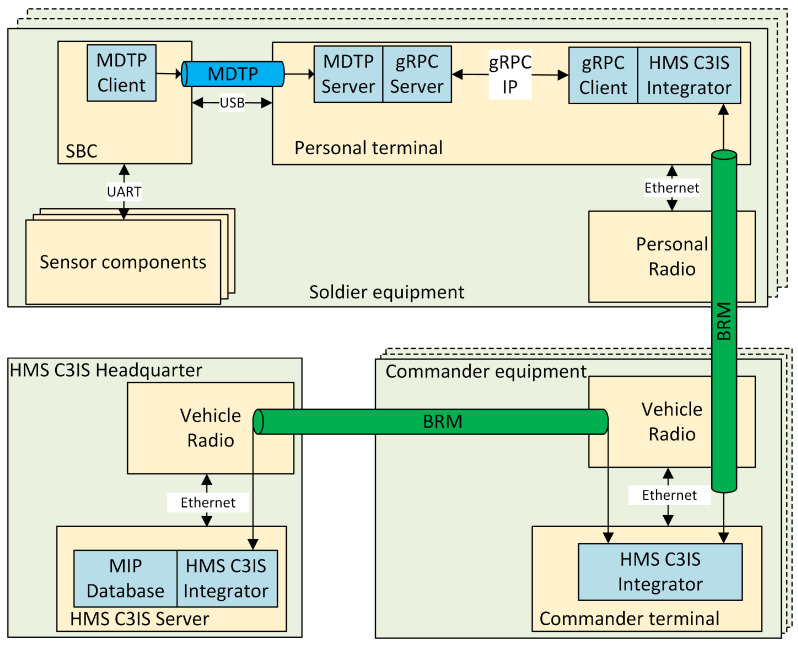
Block diagram of the Data Transmission Module (DTM).

The block diagram of the data transmission module is shown in Figure 5. Data from the measurement module are transmitted using the MDTP (DSS-MEDEVAC Data Transmission Protocol) protocol, which ensures control and reliable data transmission. This protocol allows for the determination of which parameters and signals the registration process will activate—with the possibility of modification during the registration process—to verify the correctness of the MM operations, and to control data transmission (reading the data waiting to be transmitted and its transfer). Software supporting the MDTP protocol is installed in the personal terminal and in the SBC. The received measurement data in the personal terminal are processed via the Google Remote Procedure Calls (gRPC) and are sent to the integrator with the C3IS HMS. The gRPCs are also used by the medical battlefield monitoring portal (run with the Visualization Module) to control the MM by turning on and off the recording of relevant vital signs and biomedical signals.

**Figure 6:**
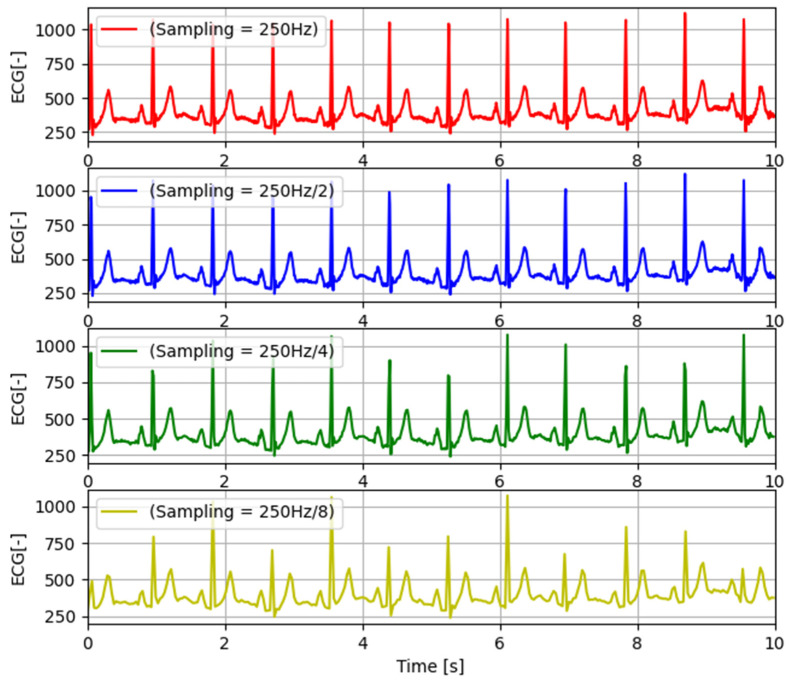
Graphical visualization of decimation of the recorded ECG signal.

Data from the integrator are then transmitted over the tactical communication network using the integrator’s Battlefield Replication Mechanism (BRM), which is the main part of the Polish HMS C3IS JASMINE system (TELDAT Sp. z o.o. sp.k., Bydgoszcz, Poland) [21] and the integral part of the mechanism for assessing reliable replication of data in accordance with the Multilateral Interoperability Program (MIP) supported by NATO and non-NATO command and control systems. The MDTP server installed in the personal terminal is also able to handle the transmission of biomedical signals (listed in Table 2). However, these signals are not distributed via the BRM; rather, the TCP/IP protocol stack is used to deliver them to the VM. Transfer of multiple biomedical signals simultaneously can overload radio links. Therefore, the MDTP server has the ability to decimate the transmitted signals. An example of the decimation possibility of a selected biomedical signal (ECG) with the highest dynamics of changes is presented in Figure 6. We used a simple decimation in which some data samples are omitted. Our system uses 4 levels of decimation (2-, 4-, 6-, and 8-fold). As can be seen in the presented charts, the characteristic features of the signal are reproduced quite well for the first two levels of decimation, while in the case of the signal in which every eighth sample is sent, it is still possible to observe the signal, but much of the details required to correctly assess the patient’s condition are not available.

**Figure 7:**
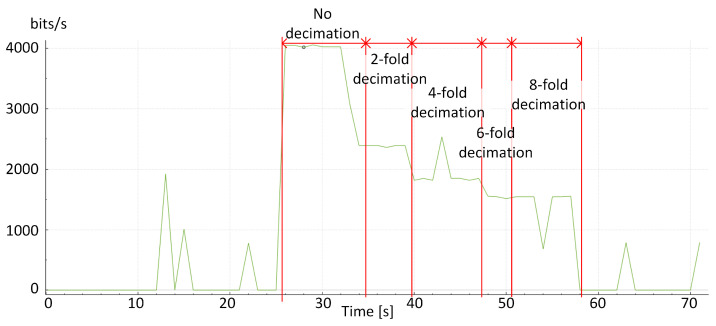
Volume of the data stream (in bits/s) of the ECG signal with selected decimations (triggered at successive moments in time).

Figure 7 shows the effect of decimation of the ECG signal on the volume of the data stream transmitted over the radio link. A significant decrease in the data stream volume with successive signal decimation can be observed. This makes it possible to transmit biomedical signals even over radio links with very low bandwidth.

### 3.4. Data Analysis Subsystem

The Analysis and Inference Module (AIM) is built as an application responsible for processing selected vital signs of the monitored soldier and, based on them, determining triage parameters that provide information about the condition of the monitored soldier and the possible need for evacuation. The necessary data including HR, RR, SpO_2_, and SBP vital signs are delivered to the AIM module from DCM via the high-performance gRPC protocol. The AIM acts as a gRPC server which is responsible for receiving incoming data streams containing vital signs values, sending them to special independent functions responsible for determining triage levels, triage reliability, and the soldier’s chance of survival and finally sending the calculated triage level to TVAM.

The developed triage algorithm described in [23], which maps different types of wounds, takes into account four basic parameters based on which it presents the system operator with an assessment of the soldier’s health condition in the following convention: blue (B), red (R), yellow (Y), and green (G).

The system has the ability to reduce the reliability of the assessment of the wounded soldier’s condition after detecting abnormalities in the measurement of a given vital sign, but it is not possible to increase it if subsequent parameters are correct. In other words, in subsequent decision branches, the algorithm may maintain or possibly increase the risk category. The order in which vital signs are considered, are determined as follows: RR-HR-SBP-SpO_2_. Such an approach is based on the expected reliability of measurements under field conditions, rather than their validity. The explanation of the adopted order of consideration of vital signs is as follows:

- HR—the highest priority, without this vital sign the system cannot function;
- RR—a vital sign that quickly reacts to changes in the wounded soldier’s condition—compensation/decompensation or lack of breathing may be caused by the soldier’s position, and the airway can be easily unblocked, and breathing can be restored;
- SBP—a vital sign secondary to the above parameters, responds “with delay”;
- SpO_2_—a vital sign with less reliability, depending on many factors.

The main algorithm for assessing a soldier’s health status and determining the triage level is shown in Algorithm 1. It maps four parameters: RR, HR, SBP, and SpO_2_ to one of the four colors: G, Y, R, or B. During the development of the algorithm after clinical trials, it was determined that typical reference values for the algorithm are as follows: 9–20 respirations per minute (rpm) for RR, 50–110 beats per minute (bpm) for HR, 100–180 mm of mercury (mmHg) for SBP, and >=94% for SpO_2_. This means that if the vital signs fall within the indicated ranges, the algorithm determines the triage color as green. The algorithm also indicates reference critical values (RRctitical, HRcritical, SBPcritical, and SpO_2_Critical), for which, depending on the combination of their values, the red color of the triage is indicated. In other cases, yellow or blue is selected. However, the range of these values has not been made public due to the protection of intellectual values. The operation of the algorithm is divided into four stages, in which RR, HR, SBP, and SpO_2_ vital signs are checked sequentially and classified into a reference or critical reference range. Each subsequent stage (except the first) is affected by the triage color determined in the previous stage. It should be noted that only in the second stage can the triage color be identified as blue (for HR = 0 bpm), which indicates the absence of vital functions of the monitored soldier. This algorithm is described in detail in [1] and is included in this paper for consistency in the description of the full version of the DSS-MEDEVAC system.
**Algorithm 1** The main algorithm for determining the level of triage**Input:**   Measured values of **RR**, **HR**, **SBP** and **SpO_2_**.   Personalized reference and critical values of the vital signs for each soldier.   Typical reference values:      **RRref**: 9–20 rpm,      **HRref**: 50–110 bpm,      **SBPref**: 100–180 mmHg,      **SpO_2_ref**: >=94%.   **RRctitical**, **HRcritical**, **SBPcritical**, **SpO_2_critical** **Output:**   **triage** **Steps:**  1. Read **RR**  2.   **if** RR == **RRref then**  3.   |        **triage** ← (Green)  4.   **else if RR** ! = **RRctitical then**  5.   |        **triage** ← (Yellow)  6.   **else**  7.   |        **triage** ← (Red)  8.   **end**  9. Read **HR** 10.   if **HR** == 0 then 11.   |        **triage** ← (Blue) 12.   **end** 13.   **if triage** == (Green) **then** 14.   |        **if HR** == **HRref then** 15.   |        |        **triage** ← (Green) 16.   |        **else if HR** ! = **HRcritical then** 17.   |        |        **triage** ← (Yellow) 18.   |        **else** 19.   |        |        **triage** ← (Red) 20.   |        **end** 21.   **else if triage** == (Yellow) **then** 22.   |        **if HR** == **HRref** or **HR** ! = **HRcritical then** 23.   |        |        **triage** ← (Yellow) 24.   |        **else** 25.   |        |        **triage** ← (Red) 26.   |        **end** 27.   **else** 28.   |        **triage** ← (Red) 29.   **end** 30. Read **SBP** 31.   **if triage** == (Green) **then** 32.   |        **if SBP** == **SBPref then** 33.   |        |        **triage** ← (Green) 34.   |        **else if SBP** ! = **SBPcritical then** 35.   |        |        **triage** ← (Yellow) 36.   |        **else** 37.   |        |        **triage** ← (Red) 38.   |        **end** 39.   **else if**
**triage** == (Yellow) **then** 40.   |        **if** SBP == **SBPref** or **SBP** ! = **SBPcritical**
**then** 41.   |        |        **triage** ← (Yellow) 42.   |        **else** 43.   |        |        **triage** ← (Red) 44.   |        **end** 45.   **else** 46.   |        **triage** ← (Red) 47.   **end** 48. Read **SpO_2_** 49.   **if**
**triage** == (Green) **then** 50.   |        **if** SpO_2_ == **SpO_2_ref**
**then** 51.   |        |        **triage** ← (Green) 52.   |        **else if SpO_2_** ! = **SpO_2_critical**
**then** 53.   |        |        **triage** ← (Yellow) 54.   |        **else** 55.   |        |        **triage** ← (Red) 56.   |        **end** 57.   **else if**
**triage** == (Yellow) **then** 58.   |        **if SpO_2_** == **SpO_2_ref** or **SpO_2_** ! = **SpO_2_critical**
**then** 59.   |        |        **triage** ← (Yellow) 60.   |        **else** 61.   |        |        **triage** ← (Red) 62.   |        **end** 63.   **else** 64.   |        **triage** ← (Red) 65.   **end** 66. **return triage** 67. End of the algorithm.

Based on the main algorithm for determining the level of triage, an auxiliary algorithm was defined to determine the survival chance function, which is used when all four stable vital signs (HR, RR, SBP, and SpO_2_) are available and only makes sense for a soldier who was in a state of relative immobility (for a certain period of time). Activity information from motion sensors and the GPS module are used to detect this condition. To define the chance of survival function, 7200 cases were generated, uniformly covering the entire four-dimensional parameter space. These cases were used to train two non-linear Support Vector Machine (SVM) networks and the final result was a chance of survival function that assigns a value of 0% to the blue class, a range of 1–50% to the red class, 51%-99% to the yellow class, and 100% to the green class. The need to define such a function result from the expectations related to the introduction of software supporting triage. The order of evacuation results from the color, but within the same color the value of the survival chance function may determine priority. In the subsequent phases of the system’s evolution, the chance of survival functions were redefined, and the latest version uses Euclidean distance measures in a four-dimensional parameter space. The pseudo-code for determining the chance of survival function is shown in Algorithm 2.**Algorithm 2** Chance of survival function calculation algorithm**Input:**   Measured values of **RR, HR, SBP** and **SpO_2_**.   Triage:      **Green, Blue, Yellow, Red.**   Extreme values of **RR, HR, SBP** and **SpO_2_**:      **RR_G_min, RR_G_max, RR_Y_min, RR_Y_max, RR_R_min; RR_R_max,**      **HR_G_min, HR_G_max, HR_Y_min, HR_Y_max, HR_R_min, HR_R_max,**      **SBP_G_min, SBP_G_max, SBP_Y_min, SBP_Y_max, SBP_R_min, SBP_R_max,**      **SpO_2__G_min, SpO_2__Y_min, SpO_2__R_min.** **Output:**   **Chance of survival** **Steps:**  1.   **if**
**triage** == (Green) **then**
  2.   |        **Chance of survival** ← 100  3.   **end**  4.   **if**
**triage** == (Blue) **then**
  5.   |        **Chance of survival** ← 0  6.   **end**  7.   **if**
**triage** == (Yellow) **then**
  8.   |        **if RR** > **RR_G_max**
**then**  9.   |        |        **DRR = (RR-RR_G_max)/(RR_Y_max − RR_G_max)** 10.   |        **else if RR < RR_G_min then**
11.   |        |        **DRR = (RR_G_min − RR)/(RR_G_min − RR_Y_min)** 12.   |        **else** 13.   |        |        **DDR** = 0 14.   |        **end** 15.   |        **if HR > HR_G_max then**
16.   |        |        **DHR = (HR** − **HR_G_max)/(HR_Y_max** − **HR_G_max)** 17.   |        **else if HR < HR_G_min**
**then** 18.   |        |        **DHR = (HR_G_min** − **HR)/(HR_G_min** − **HR_Y_min)** 19.   |        **else** 20.   |        |        **DHR =** 0 21.   |        **end** 22.   |        **if SBP** > **SBP_G_max**
**then** 23.   |        |        **DSBP** = (**SBP** − **SBP_G_max**)/(**SBP_Y_max** − **SBP_G_max**) 24.   |        **else if SBP < SBP_G_min then**
25.   |        |        **DSBP** = (**SBP_G_min** − **SBP**)/(**SBP_G_min** − **SBP_Y_min**) 26.   |        **else** 27.   |        |        **DSBP** = 0 28.   |        **end**
29.   |        **if SpO_2_ < SpO_2__G_min then**
30.   |        |        **DSpO_2_ = (SpO_2__G_min − SpO_2_)/(SpO_2__G_min − SpO_2__Y_min)** 31.   |        **else** 32.   |        |        **DSpO_2_** = 0 33.   |        **end** 34.   |        **Chance of survival** ← 100 − sqrt(**DRR**^2+**DHR**^2+**DSBP**^2+**DSpO**_2_^2)/2*50 35.   **end** 36.   **if**
**triage** == (Red) **then** 37.   |        **if RR > RR_Y_max**
**then** 38.   |        |        **DRR** = (**RR-RR_Y_max**)/(**RR_R_max** − **RR_Y_max**) 39.   |        **else if RR** < **RR_Y_min then**
40.   |        |        **DRR** = (**RR_Y_min** − **RR**)/(**RR_Y_min** − **RR_R_min**) 41.   |        **else** 42.   |        |        **DDR** = 0 43.   |        **end** 44.   |        **if HR** > **HR_Y_max then**
45.   |        |        **DHR** = (HR − HR_Y_max)/(HR_R_max − HR_Y_max) 46.   |        **else if HR **< **HR_Y_min**
**then** 47.   |        |        **DHR** = (**HR_Y_min** − **HR**)/(**HR_Y_min** − **HR_R_min**) 48.   |        **else** 49.   |        |        **DHR** = 0 50.   |        **end** 51.   |        **if SBP** > **SBP_Y_max**
**then** 52.   |        |        **DSBP** = (**SBP** − **SBP_Y_max**)/(**SBP_R_max** − **SBP_Y_max**) 53.   |        **else if SBP < SBP_Y_min then**
54.   |        |        **DSBP** = (**SBP_Y_min** − **SBP**)/(**SBP_Y_min** − **SBP_R_min**) 55.   |        **else** 56.   |        |        **DSBP** = 0 57.   |        **end**
58.   |        **if SpO_2_** < **SpO_2__Y_min then**
59.   |        |        **DSpO_2_ = (SpO2_Y_min − SpO_2_)/(SpO_2__Y_min − SpO_2__R_min)** 60.   |        **else** 61.   |        |        **DSpO_2_** = 0 62.   |        **end** 63.   |        **Chance of survival** ← 50 − sqrt(**DRR**^2+**DHR**^2+**DSBP**^2+**DSpO_2_**^2)/2*50 64.   **end** 65.   **if** (0 < **Chance of survival** < 1) **then** 66.   |        **Chance of survival** ← 1 67.   **end** 68.   **return Chance of survival** 69.   End of the algorithm.

The input parameters of the chance of survival calculation algorithm, as presented in Algorithm 2, denote threshold values determining the assigned color of a triage, where the following is true:

- Parameters starting with RR refer to respiratory rate, HR to heart rate, SBP to systolic blood pressure, and SpO_2_ to blood oxygen saturation;
- The designation Gmin and Gmax represents the lower and upper limits of the values of a given parameter assigned to the green color;
- The designation Ymin and Ymax represent the lower and upper limits of the values of a given parameter assigned to the yellow color;
- The designation Rmin and Rmax represent the lower and upper limits of the values of a given parameter assigned to the red color.
- The variables utilized in the program have the following meanings:
- DRR: Normalized distance of the current respiratory rate value from the boundary between the green and yellow colors if the triage color is yellow, or the distance from the boundary between the red and yellow colors if the triage color is red;
- DHR: Correspondingly for heart rate;
- DSBP: Correspondingly for systolic blood pressure;
- DSpO_2_: Correspondingly for blood oxygen saturation.

**Figure 8:**
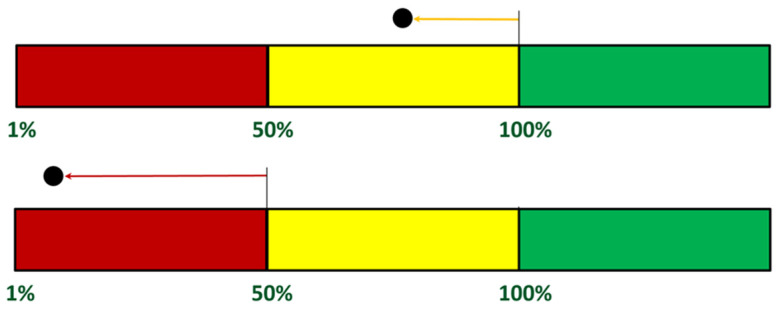
Chance of survival function when triage color is yellow (**top**) and red (**bottom**)—The colors indicate the outcome of the triage: green if the probability of survival is 100%, yellow if the probability of survival is between 50% and 100%, red if the probability of survival is between 1% and 50%.

Lines 1–6 of the code implement the assignment of chance of survival values corresponding to the green color (100%) or the blue color (0%). In lines 7–35, the chance of survival is calculated when the triage color is yellow, and in lines 36–64, when the triage color is red. In lines 65 and 66, an approximation of the survival probability value to 1% is implemented when the calculated value is less than 1%. Both parts of the algorithm, i.e., lines 7–35 and lines 36–64, are analogous. Firstly, normalized distances from the boundaries between the respective green and yellow colors or red and yellow colors are calculated for individual parameters. Subsequently, the resultant distance and survival probability function are computed: lines 34 or 63. The chance of survival function uses distance measures in an adaptively normalized feature space. In other words, when the triage color is yellow, it is the distance of the case under consideration—in the space of four diagnostic parameters—from the decision boundary separating yellow from green normalized to the distance dividing the green–yellow and yellow–red decision boundaries and is expressed as a percentage, as illustrated in Figure 8.

It should be mentioned that, on the battlefield, one or more sensors may be disconnected or damaged [24,25]. This may result from signal being cut off or strong interference preventing correct parameter determination [24]. Our system takes into account the possibility of cutting off the sensors, which are taken into account in the reliability of the calculated triage level. When all four vital signs values are available, the full algorithm is used and the chance of survival function is additionally calculated, and the reliability of the calculated triage level is 100%. In the absence of signals from one or more sensors, the survival chance function is not calculated, and the reliability is reduced accordingly. The DCM module is responsible for controlling the availability of sensor signals and vital signs. If any of the required medical parameters are missing, the DCM does not request a triage determination from the Analysis and Inference Module and Algorithm 1 and Algorithm 2 are not used to calculate triage level and chance of survival value. In addition, no new data are sent to the AIM until all required parameters are available.

Since the DSS-MEDEVAC system should handle up to 1000 soldiers at a time, the calculation of the trunk level should be efficient. The performance of triaging multiple soldiers monitored via the AIM depends on the processor used in the server performing calculations. For a machine equipped with an Intel Xeon Silver 4210 2.2 GHz processor (Intel Corporation, Santa Clara, CA, USA) and 64 GB ECC DDR4 RAM (Kingston Technology Corporation, Fountain Valley, CA, USA), determining the triage for 10,000 soldiers took less than 6 s. Taking into account sending the results to TVAM, the time increased to < 1 min.

### 3.5. Visualisation Subsystem

**Figure 9:**
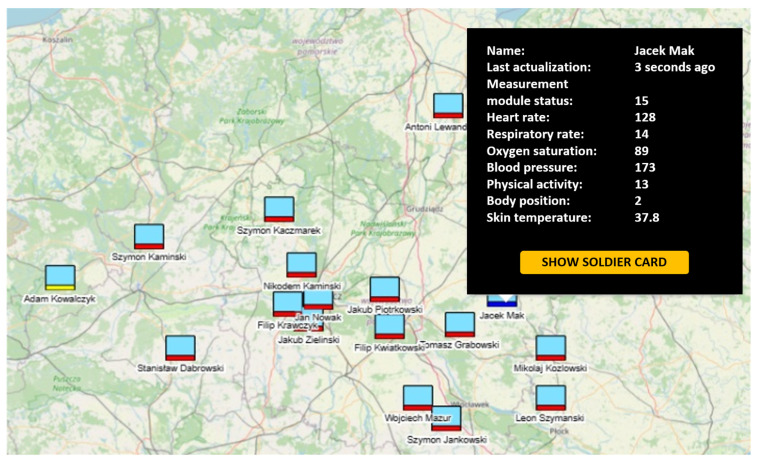
VM web portal—map view with the location of the monitored soldiers and the vital signs of the selected soldier (the light blue rectangle is a NATO tactical symbol depicting the location of a single soldier on the map; the colored rectangle under the soldier’s symbol indicates the triage colors: red, yellow and blue).

Information on monitored soldiers is saved in the HMS C3IS JASMINE system database (MIP-based) extended with medical information. In order to present the collected information to the Medical Support Group (MSG) operator responsible for medical evacuation from the danger zone, a special web-based portal was prepared. The portal allows access to detailed information collected form the MM on monitored soldiers. The software is divided into three modules responsible for handling the various data available in the DSS-MEDEVAC system. The Visualization Module (VM) is a module that allows for the display of a list of monitored soldiers and their location on the map in the portal as shown in Figure 9. triage colors are also drawn on the map in the form of a marker of a specific color under the symbol corresponding to the individual soldier on the map (an extension of NATO military symbols). Any triage result that would require MSG intervention (in red) is displayed in a separate tab as an alert that must be handled by the operator. For the selected icon representing a single soldier, information about the set of recorded vital signs is highlighted. Values are provided without units so as not to obscure the map—pointing to the icon of a soldier’s card shows all the details of the observed signs, i.e., values, units, and the quality of measurement of a given vital sign.

**Figure 10:**
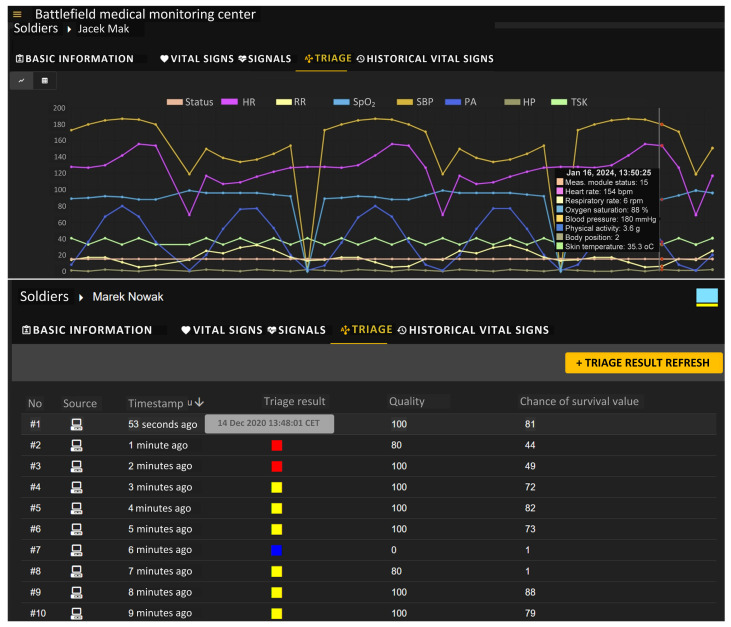
VM web portal—Current values and historical values of vital signs registered for selected soldier (upper picture) and current and historical triage colors (bottom picture); the colors on the top graph are explained in the legend; in the “Triage results” column (bottom picture), the colors of the triage (red, yellow and blue) are placed; the computer icon in the “Source” column indicates that the triage result was determined by the DSS-MEDEVAC system.

The Visualization Module allows the operator to view value changes in measured vital signs of individual soldiers as well as current and historical triage colors suggested by the system, as shown in Figure 10. In addition to the triage color, measurements’ reliability and the survival function’s value are also presented. In the figure, we can see that the system shows many more parameters than those used by the algorithm for determining the triage level. In addition to the RR, HR, SpO_2_, and SBP values (used by Algorithm 1 and Algorithm 2), the status of the measurement module, physical activity level, body position, and skin temperature are shown. It can also be noted that the resolution of the display of vital signs and triage results is 1 min, which is related to the need to minimize the traffic transmitted via tactical communication systems between the HMS C3IS JASMINE system nodes. The quality of the signals in our system is determined by the measurement module using signal processing mechanisms located in the SBC (sensor integrator). This mechanism evaluates whether a particular vital sign can be determined (calculated) from the noisy and distorted signal. The value of each parameter (e.g., HR, which is calculated from the ECG signal), along with the measurement quality assessment (in values from 0 to 100) is provided to the analysis and inference module for addition to the chance of survival values and triage level calculated according to the indicated algorithms. Thus, the values from 0 to 100 representing the “Quality” column in Figure 10 does not mean the quality of the signal, but an estimate of the level of confidence in the vital signs’ values determined from the recorded signals.

**Figure 11:**
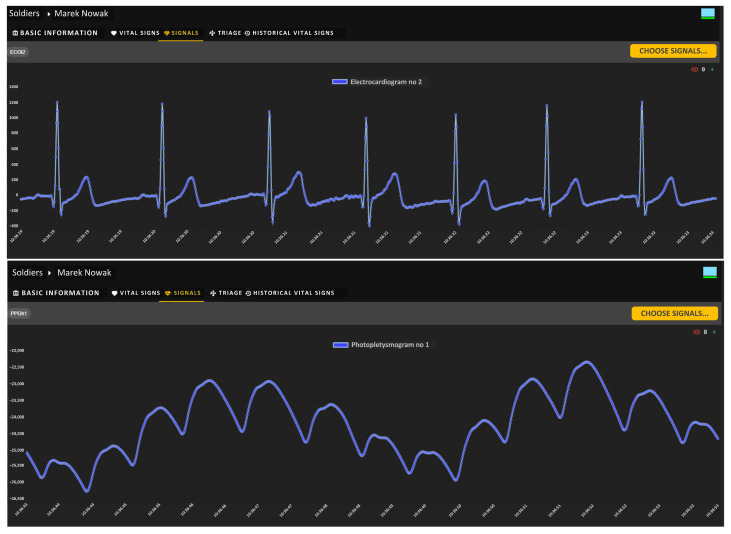
VM portal—graphical charts of ECG (**upper**) and PPG (**bottom**) signals recorded via sensors.

Using the Biomedical Signals Visualization Module, it is possible to view on-demand the biomedical signals recorded via individual sensors of a monitored soldier. Figure 11 shows sample waveforms of ECG and PPG signals of the selected soldier under testing. Both signals were delivered to the soldiers’ health monitoring center via the tactical communications network. In this scenario, signal waveforms were not decimated, as indicated by the icon with the number 0 placed in the upper right corner of each window. The medical team can remotely evaluate the shape of both signals, which are presented as a function of time.

## 4. Testing Environment, Validation, and Field Tests Results Discussion

### 4.1. Materials and Methods

The DSS-MEDEVAC system was subjected to detailed testing to validate its usability and field applicability. A number of capabilities of the system were tested, including:

- Continuous monitoring of selected soldiers’ vital signs and presenting them to medical staff;
- Displaying the current medical situation of monitored soldiers in the form of alerts on the need for medical evacuation;
- Support of medical personnel in observing selected vital signs and biomedical signals on-demand;
- Support of medical personnel in automatic assessment of soldiers’ health status and alerting in cases requiring medical evacuation;
- Support of medical personnel in automatic filling and distribution of the TC3 card;
- Support of commanders in alerting on the health status of subordinate soldiers.

In addition, carried out tests aimed at assessing the stability of the developed applications and to verify the correctness of the transfer of individual vital signs values and biomedical signal data between system components.

The system was tested in two stages. The first stage was aimed at validating the components of the system and their interaction with each other. To validate the system in this stage, predetermined conditions for system operation were assumed. In particular, vital sign test vectors were established that, when delivered correctly to the Analysis and Inference Module, enforced known triage results. In the second verification stage, the system’s performance tests were performed under field conditions on a group of soldiers (30 volunteers) performing typical tactical tasks. Due to ethical considerations, the threat to the life of soldiers was enforced artificially. In order to obtain the reference values of systolic blood pressure of soldiers during the action, in addition to the sensors of the measurement module, a portable cuff-based device for Ambulatory Blood Pressure Monitoring (ABPM) was used.

Tests in the second stage were conducted according to the following scheme:

(1) Test preparation phase (standing position, connecting the pulse oximeter to the finger, validation of vital signs values and biomedical signals graphs displayed in the portal of the visualization module, a prone position or back—normal breathing, rapid breathing (about 25 breaths/min), apnea (30 s), standing position, squats);
(2) The main phase of field tests (march along a designated route, continuous running along a designated route, crawling, standing in place—upright position, march along a designated rout, validation of vital signs values and biomedical signal graphs displayed in the portal of the visualization module);
(3) Final phase of testing (standing position, connecting the pulse oximeter to the finger, validation of vital signs values and biomedical signals graphs displayed in the portal of the visualization module).

### 4.2. Testbed Description

**Figure 12:**
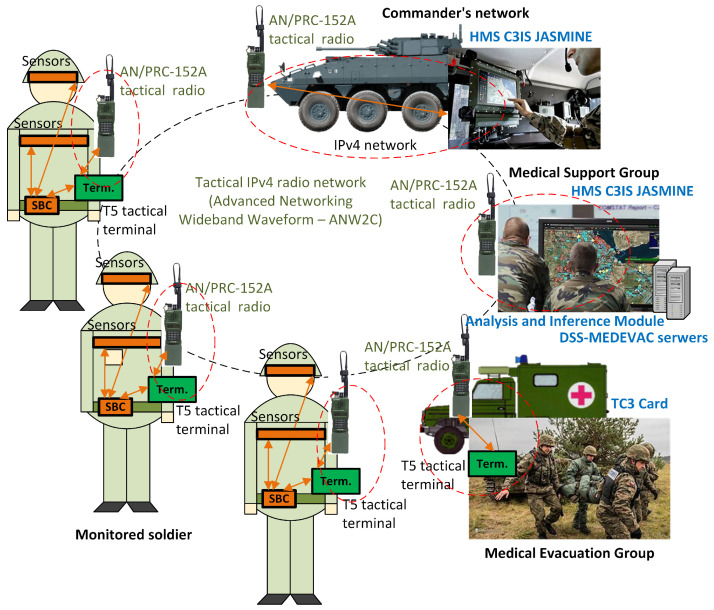
Structure of the testing environment (colors in the figure mean: orange rectangles—measurement module devices, orange dotted lines—personal and local communication networks, dark green—tactical terminal, orange arrows—information flow between soldier’s equipment, light green dotted line—tactical radio network).

A diagram of the testing environment developed and used for testing is shown in Figure 12. The environment includes all the developed components of the DSS-MEDVEAC system. The individual elements communicate via a tactical network with ANW2C waveform using the AN/PRC-152A combat radios manufactured by the Harris Corporation (Melbourne, FL, USA). The radios operate with a power level of 0.5 W, at 350.8 MHz in the 1.2 MHz band. Connected to the radio network are the soldiers’ personal equipment, the terminal that is the equipment of the commander’s vehicle, the management center with the servers of the DSS-MEDVEAC system, as well as the equipment of the vehicle of the Medical Evacuation Group. Communication in the system is carried out using IPv4 protocol.

**Figure 13:**
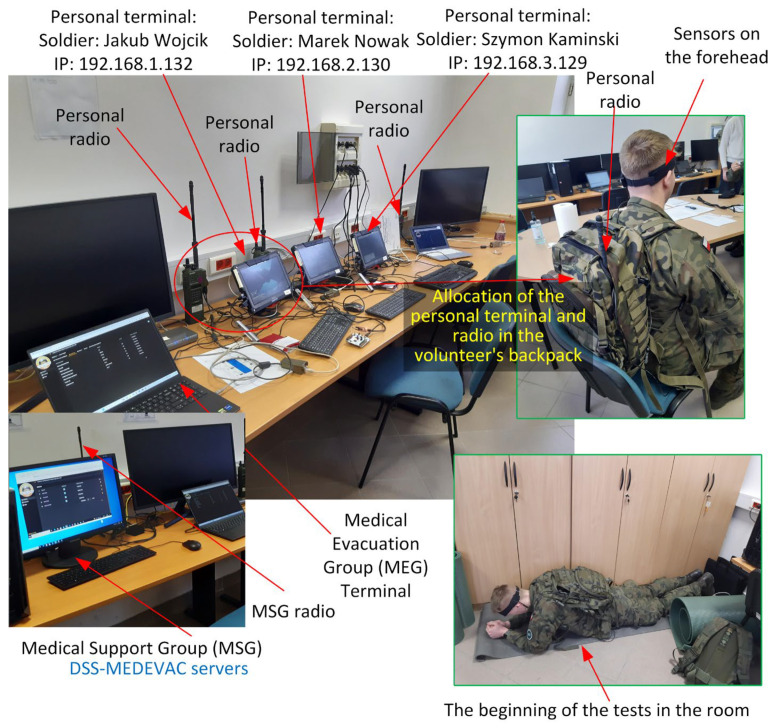
DSS-MEDEVAC system testbed—prepared devices for the field tests (font colors indicate: black—description of the DSS-MEDEVAC system test bed components, blue—description of the system’s hardware equipment, yellow—description of the equipment of the volunteer being tested; green framed photos—photos of the selected field tests).

Figure 13 shows a photo of the DSS-MEDEVAC system demonstrator in the initial testing phase, where the system’s devices are being prepared for use by soldiers during field tests. Visible are the soldier’s personal terminals, the Medical Evacuation Group terminal, the sensor forehead band, personal radios and the battlefield Medical Support Group servers. Also visible is the first phase of tests carried out by a volunteer soldier.

### 4.3. Results Discussion

**Figure 14:**
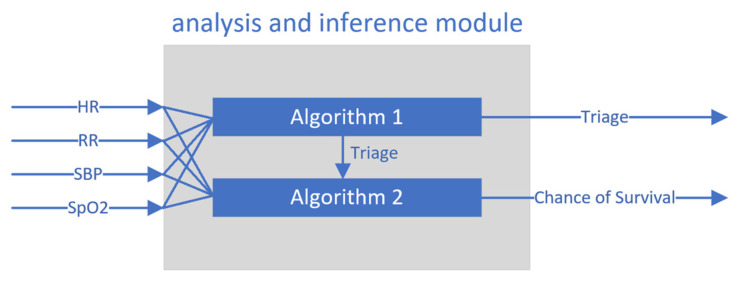
Block diagram for determining triage and chance of survival via the AIM.

Figure 14 shows a block diagram illustrating the operation of the AIM where Algorithm 1 and Algorithm 2 are implemented. For each algorithm, the vital signs such as HR, RR, SBP, and SpO_2_ are provided as input, while the output is calculated successively with the triage levels and colors (Blue, Red, Yellow, and Green) and the values of the chance of survival function. It should be noted that a prerequisite for the determination of the chance of survival function is that the triage levels must first be determined.

The triage algorithm presented in Algorithm 1 is based on the vital sign parameters provided. If the vital parameters are equal to the reference parameters, then a green triage color is determined. If the HR parameter is 0, then the triage shows blue. In other cases, the value of triage (Red, Yellow, or Green) is calculated with a special algorithm that takes into account the ranges of received vital signs values individually. The algorithm of the chance of survival function shown in Algorithm 2 is based on the determined triage levels and, in addition, on the HR, RR, SBP, and SpO_2_ vital sign values. In the simplest cases, if the triage value is green, then the chance of survival is 100, while for a blue triage color, the chance of survival is 0. In other cases, the algorithm determines the value of the chance of survival function taking into account all the values provided (triage and vital signs).

**Figure 15:**
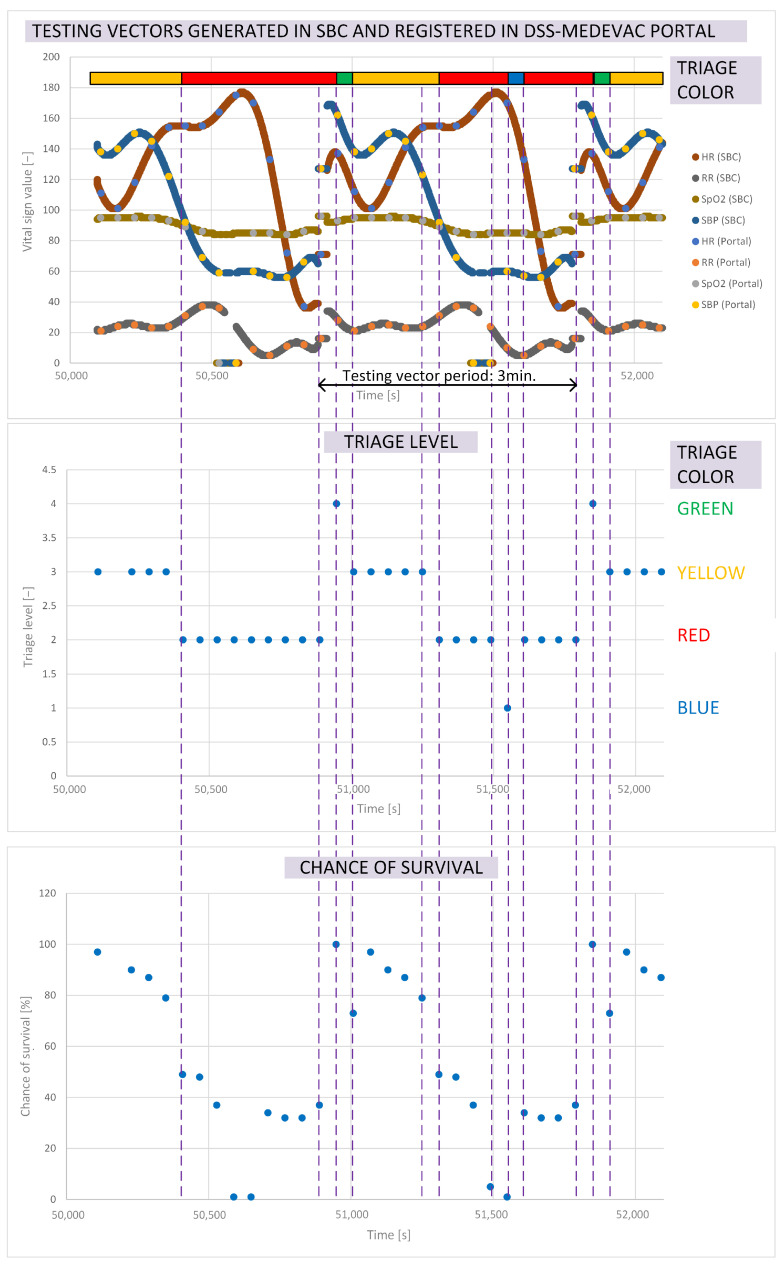
Expected response of the analysis and inference module to reference values of vital signs.

As previously mentioned, a series of vital sign test vectors were used during the first stage of the DSS-MEDEVAC system performance test. Figure 15 shows three inter-related graphs depicting the response of individual system components to one of the test vectors. The test vector chosen for presentation in this paper was one in which HR, RR, SpO_2_, and SBP vital signs were generated using sensor emulators (recorded in the SBC of one soldier), representing such forcing for Algorithm 1 and Algorithm 2 that the algorithms responded by indicating all possible levels (colors) of the triage and chance of survival functions of the emulated soldier. The length of the test vector was 3 min. For the first minute of the test vector, the values of the generated vital signs were determined in such a way that the system responded with the triage level specified by the red color. The test vector is repeated periodically. Figure 15 indicates the period of vital signs generation, where the beginning of the period shows 1 min fixed values of each vital sign.

The top image of Figure 15 shows superimposed graphs of HR, RR, SpO_2_, and SBP vital sign values delivered from the single-board microcomputer (SBC) to the soldier’s personal terminal (labeled SBC) and the values of these parameters transferred via the HMS to both the analysis and inference module and the visualization module, and presented via the battlefield medical monitoring portal (labeled Portal). The values of the vital signs are defined in the units specified in Table 1; hence, they are not indicated in the chart. At the beginning of the analysis, it can be seen that vital signs’ values labeled ‘SBC’ are transmitted to the personal terminal with a high resolution, determined via the algorithms that process sensor signals (1 s resolution). In contrast, vital signs values labeled ‘Portal’ reach the AIM module with a resolution of 1 min. After the initial vital sign values that forced the triage level to the red color, there is a period of about 1 min in which the vital signs values are at a level indicating that the monitoring subject does not need medical attention. The triage color is then indicated as green. After this time, the RR value suddenly drops, and HR and SBP rise to a level that could indicate serious medical problems, resulting in the triage being marked with a yellow color. At some point, the values of HR and SpO_2_ begin to drop significantly, and SBP begins to rise significantly, exceeding critical values, resulting in a medical alert and marking the triage with a red color (need for emergency medical evacuation). At any time, medical personnel can check in the DSS-MEDEVAC system the detailed values of the measured vital signs of the monitored soldier (and additionally the level of physical activity and body position), as well as the biomedical signals recorded via the sensors to confirm the indicated triage color. The triage results presented by the system do not replace the decision of medical personnel to evacuate the soldier, but they do enforce the need to confirm the life-threatening condition of the victims. The medical evacuation group (MEG) carrying out rescue operations for a wounded soldier on the battlefield can change the triage color manually in the DSS-MEDEVAC system after directly assessing the soldier’s condition. In the graph, we can also see the blue color of the triage, which is the result of insufficient quality of vital signs measurements. The quality of biomedical signals measurements realized via the sensors is evaluated via the measurement module.

The triage levels to which the colors shown by the visualization module correspond are indicated in the middle graph of Figure 15, while the bottom graph presents the values of the survival chance function determined with Algorithm 2. It can be seen that, for the green color of the triage, the value of the survival function is 100%, for the yellow color of the triage it drops to the level of 80%, while for the red level of the triage it drops below 50% for critical values of measured vital signs—even to 0%. It should be emphasized that, in particular, the indication of a survival function value equal to 0% must be confirmed by first responders or paramedics to determine the final triage status of the victim.

The second stage of performance evaluation of the DSS-MEDEVAC system was carried out under field conditions described in Section 4.2. Table 3 shows selected triage levels and colors as well as chance of survival function values calculated for the measured vital signs during field testing. The results presented here demonstrate the performance of Algorithm 1 and Algorithm 2 for instantaneous values of vital signs measured via the measurement modules of 13 different soldiers. For the soldier numbered 4 and 8, Algorithm 1 indicates a triage level of 4 (Green) since all vital parameters are either within reference values (green in Table 3) or not within reference critical values (red in Table 3). When the triage level is 4 (Green), the chance of survival function always indicates a value of 100%. If the value of RR is 0 rpm, it can mean either the lack of RR measurements provided via the sensor of the measurement module (sensor can be damaged or disconnected), or the death of the soldier. Therefore, we cannot unambiguously determine the level of triage in such a case. Such a value of RR, however, is relevant when determining the value of the chance of survival function. The critical vital sign for determining the triage for a given soldier is the HR for which, according to Algorithm 1, a value of 0 bpm means lack of human vital functions (the triage value is 1 (Blue)). However, this is not the case in Table 3, since testing on live humans (during field tests) was impossible to obtain an HR equal to 0 bpm. For soldiers 1, 5, 9, 12, and 13, the triage levels are 3 (Yellow) because some of the vital signs values are not in the reference or critical ranges. For the remaining soldiers, i.e., 2, 3, 6, 7, 10, and 11, a triage of level 2 (Red) was obtained, as some of the vital signs values are within the critical reference range. These values are marked in red in Table 3. In the case of soldier 10, a high value of HR = 161 bpm was recorded. This value is achievable under real conditions, such as high exertion (cardio exercise) or stress (combat operations). For the soldier tested, this value was achieved by forcing the soldier to run with a heavy load to simulate difficult combat conditions. Other critical SpO_2_ values were forced by loosely attaching a sensor to measure blood oxygen saturation. Such an action was intentional, in order to check the correctness of Algorithm 1.

**Figure 16:**
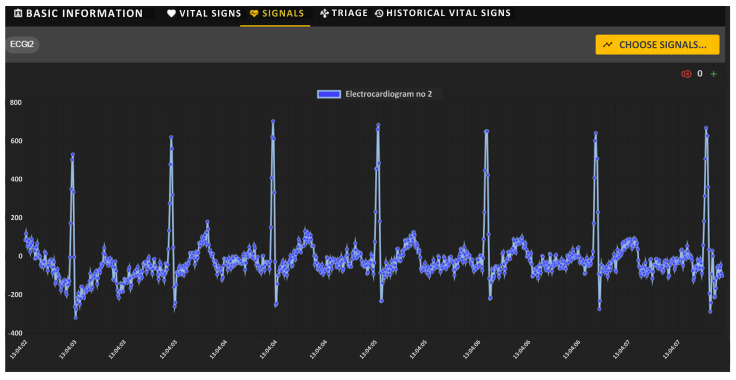
ECG signal recorded for a sample soldier under test.

As confirmation of the correctness of our system’s operation, the following figures show sample biomedical signals, observed remotely, of a selected soldier performing typical tactical activities. Figure 16 shows a sample ECG biomedical signal recorded via one soldier’s sensors, observed with the visualization module. Analyzing the shape of the ECG signal allows medical personnel to approximate the levels and location of the ECG waves, and therefore allows them to assess the current state of the soldier’s cardiovascular system.

**Figure 17:**
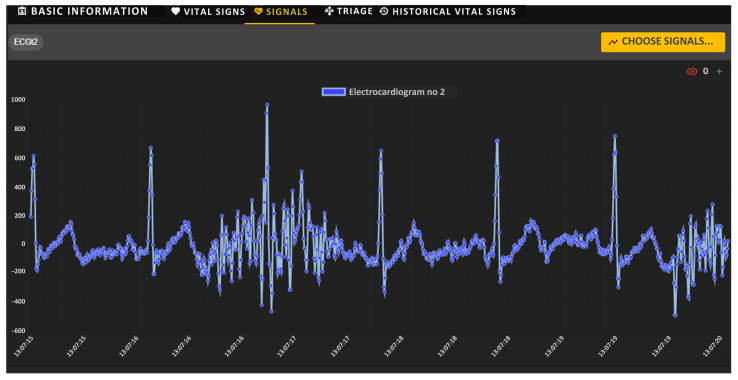
ECG signal recorded for a sample test soldier performing chest muscle contractions.

Figure 17 shows the ECG signal observed during the chest muscle contractions performed by the soldier. A corresponding signal shape response in the form of distorted waves is visible.

**Figure 18:**
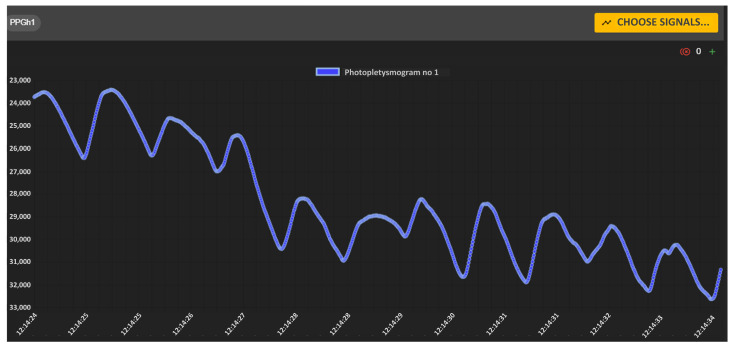
PPG signal recorded for a sample soldier under test.

Figure 18 shows the recorded PPG signal of a remotely observed soldier. The PPG signal itself is used to automatically determine the RR value, while the shape of this signal, like the ECG, can indicate the current state of the soldier’s health.

**Figure 19:**
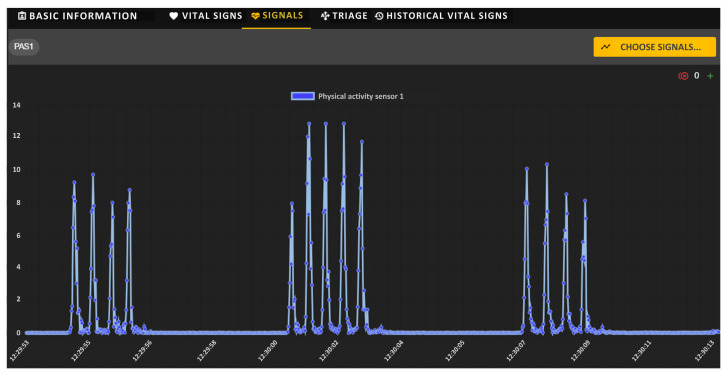
Physical activity signal for a sample soldier.

The physical activity graph of the selected soldier is shown in Figure 19. This signal provides an auxiliary factor to determine the causes in the deviations of the instantaneous values of ECG and PPG signals resulting from the soldier’s activity. The figure shows a significant periodic activity of the soldier reaching a value of 12 g (represented as a multiplier of the standard gravity g).

## 5. Conclusions and Future Work

This paper describes the architecture of a decision support system for medical personnel in the field of medical triage dedicated mainly to the armed forces implementing tactical operations. Soldiers can be continuously monitored via prepared medical sensors and, if necessary, quickly evacuated from the battlefield by military medical services. The system developed by the authors of this paper was tested under conditions similar to real tactical operations. The results of the tests showed that the assumed capabilities of determining the triage level for soldiers and alerting medical services were achieved. This paper also presents the author’s method of determining the chances of survival of soldiers, which, in addition to the typical values of vital signs recorded via the sensors, is an additional determinant of determining the color of the triage, helpful in making the final decision on the evacuation of wounded soldiers from the battlefield.

The system presented in this paper has a number of advantages. Since it is dedicated to military applications, it is much more advanced in terms of resistance to extreme field conditions. In addition, it allows integration with command-and-control systems. It offers enhanced situational awareness and real-time decision support, which is crucial in the context of battlefield medical evacuation. In addition, the implementation of a survivability function allows the triage to be optimized even before medical evacuation teams arrive at the scene. Despite its many advantages, few drawbacks to the system can also be identified. These include, for example, the need to absorb communication means for continuous transmission of measurement data to the analysis and inference module. There is, therefore, a limitation to the use of the system in incidental radio silence conditions. Nor can we remain silent about the fact that the proposed algorithm, or rather its parameters, was developed based on both medical simulations and field tests, but not in actual combat operations and only on healthy volunteers, which may lead to suboptimal system responses in a real battlefield scenario. Therefore, in the long term, there are plans to implement the system in a small tactical unit and conduct research in actual combat conditions. The biomedical signals recorded during real military operations will allow for the final tuning of the algorithm’s parameters. Naturally, this does not preclude the possibility of further research, which we plan to continue.

Further work is aimed at fully implementing the system into the armed forces. In addition, AI technology is being considered for the system. In particular, it seems reasonable to use SVMs (Support Vector Machines) and DNNs (Deep Neural Networks); however, this requires the collection of a large amount of authentic data. Human organisms are often very different, and we anticipate that future instances will arise where the recorded parameters exceed standard norms, with extreme elevations or reductions in certain parameters not resulting from injuries but rather due to stress or significant exertion. Such cases should be collected and utilized as training data for AI algorithms, which, recognizing such exceptional syndromes in a combat-active system, could make certain modifications to the chance of survival function or even the color of triage. Given the aforementioned significant variability of individual human organisms, the application of AI must be approached with particular caution.

## Figures and Tables

**Table 1:** List of vital signed measured with the measurement module of the DSS-MEDEVAC system.

Vital Sign Name	Shortcut	Data Size [Bytes]	Scope of Change	Measurement Sensor
Heart Rate	HR	3	20–250 [bpm]	ECG sensor
Respiratory Rate	RR	2	2–60 [rpm]	RES sensor
Oxygen Saturation	SpO_2_	2	50–100 [%]	PPG sensor
Systolic Blood Pressure	SBP	3	40–250 [mmHg]	ECG and PPG sensors
Physical Activity	PA	2	0–14 [g—standard gravity]	ACC and PAS sensors
Human Body Position	HP	2	0–2 (0—lying down pose, 1—standing, 2—unspecified)	ACC sensor
Skin Temperature	TSK	3	20–45 [°C]	Skin temperature sensor

**Table 2:** List of biomedical signals measured with sensors.

Signal Name	Shortcut	Sampling Frequency [Hz]	Data Rate [bit/s]
Electrocardiocram	ECG	200, 100, 50	6400, 3200, 1600
Photoplethysmogram	PPG	200, 100, 50, 25	6400, 3200, 1600, 800
Respiratory rate signal	RES	50, 25, 10	800, 400, 160
Accelerometer	ACC	100, 50, 25	1600, 800, 400
Physical activity signal	PAS	50, 25	800, 400

**Table 3:** The level of triage and the values of the chance of survival function for selected soldiers performing a typical tactical activity (note: life-threatening vital signs values were forced artificially—heavy physical exertion or a loose sensor)—the colors of the selected cells of the table, are explained in the text.

Soldier #	HR [bpm]	RR [rpm]	SBP [mmHg]	SpO_2_ [%]	Algorithm 1 (Triage Level and Color)	Algorithm 2 (Chance of Survival) [%]
1	133	25	123	98	3 (Yellow)	81
2	94	19	126	64	2 (Red)	34
3	148	27	108	34	2 (Red)	15
4	100	20	122	94	4 (Green)	100
5	120	30	124	92	3 (Yellow)	71
6	120	26	121	87	2 (Red)	48
7	118	0	129	45	2 (Red)	9
8	79	0	125	96	4 (Green)	100
9	96	0	132	93	3 (Yellow)	25
10	161	28	117	90	2 (Red)	47
11	98	17	150	81	2 (Red)	44
12	106	22	118	98	3 (Yellow)	95
13	117	25	138	98	3 (Yellow)	87

## Data Availability

The original contributions presented in the study are included in the article, further inquiries can be directed to the corresponding authors.

## Abbreviations

4G/5G	4th/5th Generation mobile networks
ABPM	Ambulatory Blood Pressure Monitoring
ACC	Accelerometer
AFE	Analog Front End
AI	Artificial Intelligence
AIC	Analysis and Inference Capability
AIM	Analysis and Inference Module
ANW2C	Advanced Networking Wideband Waveform
ARM	Advanced RISC Machine
BIS	Bispectral Measurement Electrodes
BMS	Battlefield Management System
BP	Body Position
BRM	Battlefield Replication Mechanism
BSVM	Biomedical Signals Visualization Module
C3IS	Command, Control, Communication, Intelligence, and Surveillance
CNN	Convolutional Neural Networks
DCM	Data Collection Module
DDR4	Double Data Rate Synchronous Dynamic Random Access Memory (version 4)
DHR	Normalized distance of the current heart rate
DRR	Normalized distance of the current respiratory rate value
DSBP	Normalized distance of the current systolic blood pressure
DSpO_2_	Normalized distance of the current blood oxygen saturation
DSS-MEDEVAC	Decision Support System for Medical Evacuation
ECC	Error Checking and Correction
ECG	Electrocardiogram
GRPC	Google Remote Procedure Calls
HMS	Headquarter Management System
HP	Human Body Position
HR	Heart Rate
IPv4	Internet Protocol version 4
LTE	Long Term Evolution
MDTP	DSS-MEDEVAC Data Transmission Protocol
MEDEVAC	Medical Evacuation
MEG	Medical Evacuation Group
MIP	Multilateral Interoperability Program
MM	Measurement Module
MSG	Medical Support Group
NAFv4	NATO Architecture Framework version 4
PA	Physical Activity
PAS	Physical Activity Signal
PAT	Pulse Arrival Time
PPG	Photoplethysmogram
RAM	Random-Access Memory
RES	Respiratory Rate Signal
RR	Respiratory Rate
SBC	Single Board Computer
SBP	Systolic Blood Pressure
SpO_2_	Oxygen saturation of the blood
SVM	Support Vector Machines
SVR	Support Vector Regression
TCCC, TC3	Tactical Combat Casualty Care
TSK	Skin Temperature
TVAM	Triage Visualization and Alarm Module
UART	Universal Asynchronous Receiver-Transmitter
USB	Universal Serial Bus
VM	Visualization Module
